# The Application of Mobile Devices for Measuring Accelerations in Rail Vehicles: Methodology and Field Research Outcomes in Tramway Transport

**DOI:** 10.3390/s25154635

**Published:** 2025-07-26

**Authors:** Michał Urbaniak, Jakub Myrcik, Martyna Juda, Jan Mandrysz

**Affiliations:** 1 Department of Transportation Engineering, Faculty of Civil and Environmental Engineering, Gdansk University of Technology, 80-233 Gdansk, Poland; s186490@student.pg.edu.pl (J.M.); s186643@student.pg.edu.pl (M.J.); 2 Department of Radiological Informatics and Statistics, Medical University of Gdansk, 80-210 Gdansk, Poland; jan.mandrysz@gumed.edu.pl

**Keywords:** tramway, accelerations, mobile measurements

## Abstract

Unbalanced accelerations occurring during tram travel have a significant impact on passenger comfort and safety, as well as on the rate of wear and tear on infrastructure and rolling stock. Ideally, these dynamic forces should be monitored continuously in real-time; however, traditional systems require high-precision accelerometers and proprietary software—investments often beyond the reach of municipally funded tram operators. To this end, as part of the research project “Accelerometer Measurements in Rail Passenger Transport Vehicles”, pilot measurement campaigns were conducted in Poland on tram lines in Gdańsk, Toruń, Bydgoszcz, and Olsztyn. Off-the-shelf smartphones equipped with MEMS accelerometers and GPS modules, running the Physics Toolbox Sensor Suite Pro app, were used. Although the research employs widely known methods, this paper addresses part of the gap in affordable real-time monitoring by demonstrating that, in the future, equipment equipped solely with consumer-grade MEMS accelerometers can deliver sufficiently accurate data in applications where high precision is not critical. This paper presents an analysis of a subset of results from the Gdańsk tram network. Lateral (*x*) and vertical (*z*) accelerations were recorded at three fixed points inside two tram models (Pesa 128NG Jazz Duo and Düwag N8C), while longitudinal accelerations were deliberately omitted at this stage due to their strong dependence on driver behavior. Raw data were exported as CSV files, processed and analyzed in R version 4.2.2, and then mapped spatially using ArcGIS cartograms. Vehicle speed was calculated both via the haversine formula—accounting for Earth’s curvature—and via a Cartesian approximation. Over the ~7 km route, both methods yielded virtually identical results, validating the simpler approach for short distances. Acceleration histograms approximated Gaussian distributions, with most values between 0.05 and 0.15 m/s^2^, and extreme values approaching 1 m/s^2^. The results demonstrate that low-cost mobile devices, after future calibration against certified accelerometers, can provide sufficiently rich data for ride-comfort assessment and show promise for cost-effective condition monitoring of both track and rolling stock. Future work will focus on optimizing the app’s data collection pipeline, refining standard-based analysis algorithms, and validating smartphone measurements against benchmark sensors.

## 1. Introduction

Monitoring the condition of rail vehicles and track infrastructure is critical for ensuring operational safety and passenger comfort [1,2,3,4]. Precise monitoring of dynamic interactions enables early fault detection and the optimization of preventive maintenance and repair strategies, thereby prolonging the service life of both rolling stock and track components [5,6,7]. Real-time monitoring systems reduce the risk of failure and lower infrastructure maintenance costs [8,9].

Unbalanced accelerations encountered during vehicle operation substantially affect both the rolling stock and the track surface [10,11]. Short, dynamic impulses detrimentally impact passenger comfort, and their intensity—dependent on operating conditions and vehicle technical parameters—has been confirmed by numerical simulations and experimental studies [12,13]. High-intensity impacts can accelerate infrastructure wear and lead to significant repair expenses [14,15].

Conventional monitoring solutions rely on specialized, high-precision accelerometers and sophisticated software, but their deployment entails substantial capital outlay—particularly challenging for municipally funded tram networks [16,17]. In response to these constraints, modern mobile technologies have emerged, enabling real-time recording of dynamic data at a fraction of the cost. Smartphones, equipped with MEMS (Microelectromechanical Systems) accelerometers and GPS (Global Positioning System) modules, can precisely geolocate rail vehicles while capturing the magnitude and characteristics of dynamic impacts [12,18]. This capability allows measurement data to be correlated with specific track segments, facilitating targeted inspection, diagnostics, and maintenance planning [9,19].

Continuous monitoring and real-time data collection methods are widely applied in high-speed rail (HSR) systems for infrastructure diagnostics. A key approach is Structural Health Monitoring (SHM), which enables long-term tracking of ballastless track slab deformation. These systems often rely on high-precision accelerometric sensors, capable of detecting subtle dynamic responses of the infrastructure under operational loads. Due to the heteroscedastic nature of SHM data—affected by dynamic train loading, electromagnetic interference, temperature fluctuations, and ongoing maintenance—advanced modeling techniques are required. One study introduced an SHM system based on fiber Bragg grating (FBG) sensors with temperature self-compensation and employed a Variational Heteroscedastic Gaussian Process (VHGP) model to improve uncertainty estimation and forecasting accuracy [20]. Another approach combined fiber optic sensors with a random forest algorithm to identify slab deformations such as warping or delamination, achieving a classification accuracy of 96.09% [21]. A third study used a train–track–bridge-coupled model and accelerometric data to correlate train dynamics with bridge-induced deformations, enabling the derivation of safety thresholds for structural changes [11].

Rail vehicles experience both primary and vibratory motion. The primary motion involves uniform movement of all points, while vibratory motion—periodic, quasi-periodic, or random—is superimposed on it [22,23].

These vibrations result from dynamic vehicle–track interactions, influenced by track geometry, elastic deformations, wheel–rail contact, and operating conditions such as speed and direction. Unbalanced accelerations arising from these interactions can lead to serious consequences, including fatigue cracks in components, increased rolling resistance, reduced infrastructure lifespan, and elevated noise emissions. They also increase the likelihood of exceeding the clearance envelope, and negatively affect both passengers and staff [22,24].

Vibrations include whole-body vibrations from the vehicle’s structure to the human body, horizontal vibrations occurring in the direction of travel and perpendicular to it, and vertical vibrations acting along the vertical axis of the human body [25,26].

Rail vehicle vibrations originate from multiple factors, including elastic deformations in vertical and horizontal planes, geometric irregularities, and the dynamic properties of the wheel–rail interface. Variations in vehicle speed and direction—whether during uniform or non-uniform motion on straight segments, curves, or transition zones—also influence vibratory behavior. Additionally, the traction system and any coupled vehicles generate dynamic forces acting on the car body. From a human-factors perspective, unbalanced accelerations can induce forced body vibrations, particularly when their frequencies coincide with the natural resonance of body parts. For vertical vibrations, resonance typically occurs between 4 and 6 Hz (affecting the spine, torso, and abdomen), whereas horizontal oscillations are critical in the 1–3 Hz range [22,27]. Consequently, ride comfort may be assessed using these vibration parameters.

Traction units typically produce vibrations in the 0–20 Hz range, with particularly high acceleration amplitudes in the 0–4 Hz, 6–12 Hz, 16–25 Hz, and 60–120 Hz bands. Prolonged exposure to such vibrations may impair cognitive function, slow reaction times, and induce motion sickness or vibration syndrome—potentially affecting memory and perception due to disruptions in brain activity [22,26].

According to the standard PNEN 12299:2009 [28], the evolution of passenger ride comfort inside rail vehicles is based on the analysis of accelerations in various directions acting on the human body. The standard defines specific comfort indices:

- Basic evaluation involves the analysis of a long-duration measurement segment with continuous data acquisition. It can be assessed using the basic method, in which measurement devices are placed on the vehicle floor at the front, middle, and rear of the train, on the inner side of the track. An alternative is the accurate method, where measurements are also taken from the vehicle floor, but allow for analysis at the additional measurement points beyond those defined in the basic method.
- Continuous evaluation accounts for short-term variations in passenger perception. Acceleration measurements are recorded from the floor of the vehicle in three orthogonal directions, capturing rapid changes in vibrational input.
- Transitional curve evaluation refers to the discomfort experienced by passengers when the rail vehicles travel through transition curves into or out of a bend. This assessment focuses on sudden changes in motion and lateral accelerations and includes the recording of roll velocity.
- Discrete event evaluation assesses the impact of short-term, noticeable fluctuations such as abrupt speed changes, vibrations, and jolts. Measurement devices for this purpose are also mounted on the floor of the transport unit.

Unbalanced accelerations are evaluated using simulation models, vibration analysis, and signal-processing techniques. Data are interpreted with specialized software for vehicle dynamics and human-response assessment. Instrumentation typically includes triaxial accelerometers, data recorders, and integrated GPS–IMU systems, often equipped for real-time diagnostics and maintenance monitoring.

A study in a major Polish city employed onboard data recorders in public-transport vehicles to monitor driver behavior and passenger comfort. The system measured root-mean-square (RMS) acceleration every three seconds and flagged values exceeding predefined thresholds (e.g., 1.5 m/s^2^ for longitudinal and lateral axes, and approximately 9.6–10.0 m/s^2^ for vertical motion). Real-time feedback guided drivers in improving their operation, while GPS-logged exceedances enabled the creation of route-maps highlighting locations of passenger discomfort [26].

In 1914, German researchers investigated the effects of vertical and lateral sinusoidal vibrations on human volunteers. They determined that ride comfort depends both on the maximum kinetic energy transferred to the body and on the rate of change of acceleration. From these findings, the ride-smoothness index (*Wz*) was developed, correlating with passenger fatigue time and subjective comfort, and ranging from “very good” to “dangerous” [22].

In 1994, a study evaluated the SB3 elastic-fastening system by measuring accelerations and forces on bogies and car bodies at various speeds. Key metrics included vehicle stability, wheel–rail interaction forces, and ride comfort—assessed via lateral, vertical, and longitudinal accelerations. The results informed decisions on increasing operational speed, ultimately contributing to a new rail-speed record in Poland. Separate values were calculated for vertical and lateral vibrations and analyzed across different speeds. Comfort was assessed using the *Wz* smoothness index and maximum allowable exposure times to vibrations. Measurements showed that travel at 220 km/h still provided very good ride comfort, and speeds up to 250 km/h on SP-type elastic fastenings were deemed safe [29].

Passenger stability is closely linked to acceleration and deceleration values. While higher acceleration improves energy efficiency and reduces travel time, it also increases the risk of imbalance for standing passengers. Studies conducted on the Tyne and Wear Metro system (UK) showed that discomfort most often occurs during rapid deceleration, initial movement, or transitions between acceleration phases. A critical factor in passenger comfort is not only the magnitude of acceleration but also jerk—the rate of change of acceleration. Maintaining smooth acceleration profiles with limited jerk values is essential for ensuring stability, comfort, and operational safety in passenger rail systems [15].

Spectrum analysis can be a useful tool for assessing acceleration measurements, as it allows visualization of acceleration amplitude within specific frequency bands. However, this type of analysis is more challenging when data are collected at non-uniform sampling frequencies [30].

Using smartphones for acceleration measurements may be useful in determining road roughness and estimating energy loss [31,32]. Although GPS signals may be unavailable in subway environments, smartphones can infer location based on acceleration patterns at certain stations [33]. Smartphones in rail systems are mostly used to determine track condition [34,35], but research on passenger comfort in China found that, compared to professional onboard systems, the relative error ranged from 2% to 10% [36].

The present study utilizes data collected during field campaigns in several Polish cities—Gdańsk, Olsztyn, Bydgoszcz, and Toruń [37]. The test sites were chosen to represent diverse infrastructure conditions, surface types, and rolling-stock fleets (see Materials and Methods). To illustrate the methodology, we focus on a case study of two tram lines in Gdańsk. Dynamic data—three-axis acceleration measurements—were analyzed, with an emphasis on lateral and vertical components; longitudinal acceleration was excluded as it is largely governed by driver behavior [38]. Data processing, analysis, and visualization were performed in R, yielding detailed plots of the raw measurements, while spatial distributions of dynamic impacts were visualized via cartograms produced in ArcGIS.

This research holds both engineering and economic significance. Affordable mobile technologies enable continuous monitoring of operational conditions and rapid identification of anomalies, supporting more efficient municipal budget management and operational-cost optimization. Nevertheless, questions remain regarding the reliability and accuracy of low-cost monitoring systems. Previous studies indicate that mobile devices can supply relevant and sufficiently precise data on dynamic impacts, and that MEMS-GPS integration permits accurate analysis of key operational parameters along an entire route [38,39].

In this paper, we present a streamlined approach for measuring unbalanced accelerations in rail vehicles using widely available mobile devices and an Android-based measurement application.

## 2. Materials and Methods

This work is based on the results of accelerometer measurements in rail-passenger transport vehicles. The aim of the field research was to better understand how unbalanced accelerations affect passenger comfort in rail transport and to compare these measurements with passengers’ subjective perceptions. The study encompassed unbalanced acceleration measurements inside Electric Multiple Units (EMU), Diesel Multiple Units (DMU), and trams. Data were collected in Bydgoszcz, Toruń, Olsztyn, and Gdańsk (in Poland), as well as on various railway lines. During the field campaigns, vehicle speed, geographic coordinates, three-axis unbalanced accelerations, and passenger comfort ratings (according to the NMV ISO PN-EN 12299:2009 scale) were recorded, yielding a dataset of over 19 GB of uncompressed data and metadata saved in CSV, TXT, and XLSX formats.

For this work, the test site where the measurements were carried out was located in Gdańsk. In this city, the tram route selected for analysis covered the section between Śródmieście SKM and Lawendowe Wzgórze. The entire tram system in Gdańsk operates on standard-gauge tracks (1435 mm) and is powered by 600 V DC supply. The selected route runs from the Śródmieście SKM stop, located in the city-center district, to the Lawendowe Wzgórze stop, situated on the border between Ujeścisko-Łostowice and Jasień district. This route is designated as line A1 in the direction toward Śródmieście SKM, and 1B in the direction toward Lawendowe Wzgórze.

The first segment of the route from Śródmieście SKM to Chełm Witosa, was opened in 2007. In 2012, the line was extended to the Łostowice Świętokrzyskie terminal, and in 2023, a further extension from Przemyska to Lawendowe Wzgórze was put into service.

Due to the layout of the Lawendowe Wzgórze terminal, which is not a loop, trams operating in this section must be bidirectional. As a result, two different tram models can be found in service on routes to Lawendowe Wzgórze, both of which are described in general terms below.

Düwag N8C are second-hand tram vehicles imported from the German cities of Dortmund and Kassel, equipped with drivers cabs at both ends. They were manufactured between 1978 and 1986 and brought to Gdańsk in 2007 from Dortmund and in 2014 from Kassel. In total, Gdańsk owns 64 units of these vehicles.

Modernizations took place in 2009, 2015, and 2017, which changed the external appearance of the trams, added air conditioning in some units, and most importantly, replaced the middle section of the vehicles from high-floor to low-floor. Some components were also moved from underneath the tram to the roof, following the design of newer rolling stock.

**Figure 1:**
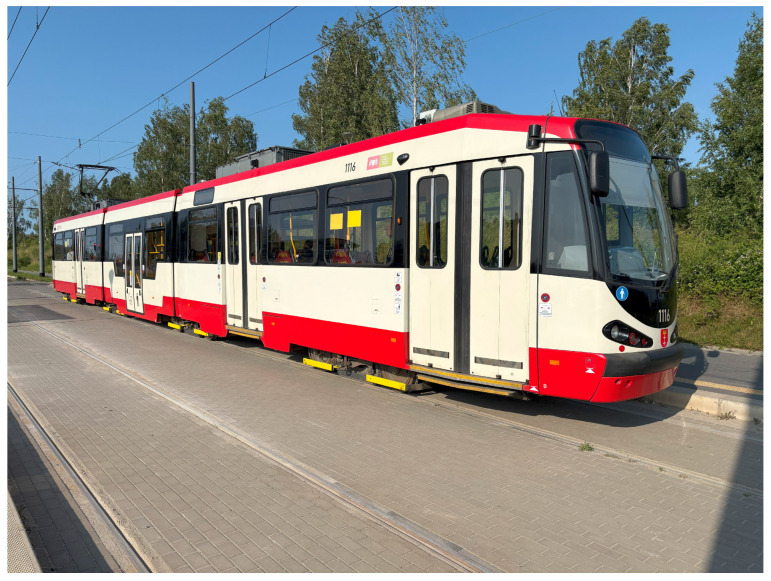
N8C tram at the Lawendowe Wzgórze stop.

In total, each vehicle has 8 bogies with 2 axles each, and the entire tram consists of 3 sections. Front and last bogies are single-section bogies and two in the middle are Jacobs bogies. The total length of the vehicle is 26.64 m, with a width of 2.33 m and a height of 3.30 m. The tram can carry 176 passengers, including 42 seated and 134 standing. The tare weight of the vehicle is 36 tons. An image of the N8C tram is shown in Figure 1.

**Figure 2:**
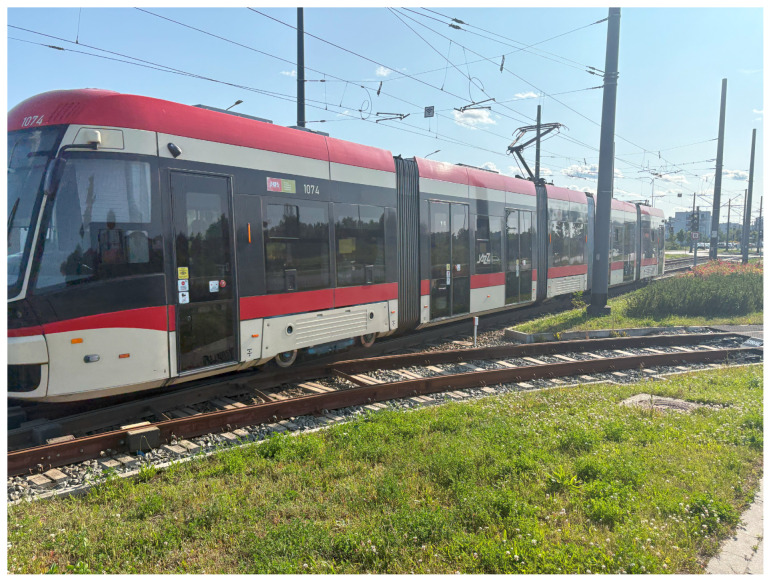
128NG tram entering the Ujeścisko stop.

The second vehicle operating on this route is the Pesa 128NG Jazz Duo tram. These trams were delivered from Pesa factory in Bydgoszcz for the first time in 2014. They are fully low-floor vehicles, even in the bogie areas, due to their independent wheel suspension design. The trams are 29.7 m long, 2.4 m wide, and 3.4 m high. They run on three bogies and are composed of five sections. The tram can carry 205 passengers, 36 seats, and 179 standing places. The unladen weight of the vehicle is 42.5 tons. Pesa 128NG is fully low floor without elevated floor above bogies. It is possible, thanks to forgoing axis and constructing each wheel used to accelerate with individual propulsive system. All bogies are single-section bogies. A photo of the 128NG tram is shown in Figure 2.

**Figure 3:**
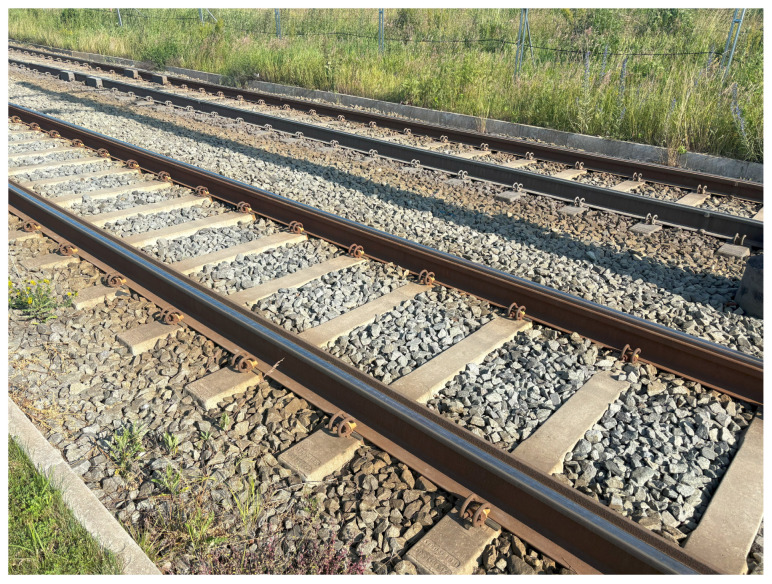
Track on the newest section of the route features a ballasted track structure and uses 60R2 rails.

Due to the varying commissioning times of individual segments of Route 1, differences in the condition of the track infrastructure can be expected along its length, which may affect the perceived level of passenger comfort. For the majority of the route, an uncovered ballasted track structure was employed, as illustrated in Figure 3. Embedded track structures were used along portions of the stops. The rails utilized along the route include standard rail type 49E1, as well as grooved rail type 60E2, the latter primarily applied in curved sections and within stop areas.

The measurements were carried out using smartphones equipped with MEMS-type sensors and the Physics Toolbox Sensor Suite Pro application [40], which includes functionalities such as gyroscope, inclinometer, oscilloscope, spectrogram, linear accelerometer, GPS receiver, and others. The primary function used for the measurements was the linear accelerometer, which enables the recording of deviations along the *x*, *y*, and *z* axes over time, expressed in m/s^2^. The *x*-axis represented lateral deviations relative to the vehicle’s direction of travel, the *y*-axis represented longitudinal deviations, and the *z*-axis represented vertical tilt relative to the vehicle’s vertical axis. The following smartphones were used for data collection: Samsung Galaxy S10e, Samsung Galaxy S6, Xiaomi Mi 10T Lite 5G, Xiaomi Redmi Note 9, Samsung Galaxy A70, Xiaomi Mi 10T, and iPhone 14 Plus. Data sampling frequency was not consistent across models. Prior to each measurement, all devices were configured to save data in local time and to use the “Quick” sampling setting, which offers the highest number of samples. However, due to hardware and firmware differences, actual sampling rates varied between models. Within a single dataset from one device, the sampling interval often fluctuated between 0 ms and 20 ms.

According to the PN-EN 12299:2009 standard, measurement devices intended for evaluating ride comfort should be rigidly mounted to the vehicle structure—typically on the floor—in locations representative of the passenger experience. These mounting positions are selected to ensure accurate recording of accelerations acting on the human body in the longitudinal, lateral, and vertical directions.

**Figure 4:**
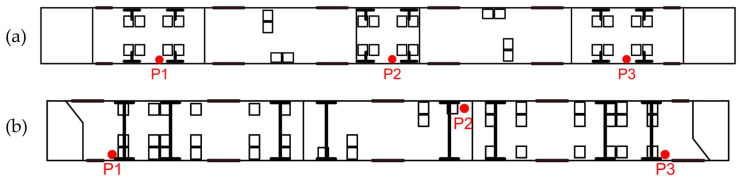
(**a**) Schematic diagram of the Pesa 128NG tram indicating the sensor measurement points, (**b**) Schematic diagram of the N8C tram indicating the sensor measurement points.

Each measurement involved collecting data from three sensor positions within the vehicle. The measurement points were distributed at the front, middle, and rear of the vehicle, as illustrated in Figure 4.

The measurements were conducted above the bogies within the vehicles. The sensor configuration varied depending on the number of passengers on board; however, the intended goal was to position all measurement points on one side of the vehicle. In the N8C tram, the second point was located on the opposite side relative to the others due to the seat placement on the right side, under which the smartphone used for measurements was positioned. Additionally, the N8C does not have a bogie located at the geometric center of the vehicle, so 1 of 2 bogies in the central section was selected for measurement.

Smartphones were placed on rubber anti-skid pads, with the top edge facing the direction of travel. Their exact positions varied by vehicle and measurement point. In the Pesa 128NG, all devices were positioned on the shelf beside the passenger seat, approximately 30 cm above the seat or at seat level, depending on travel conditions. In the Düwag N8C, units at points 1 and 3 were placed on the sandboxes beneath the first and last bogies (located under the seats). Due to the absence of a sandbox in the middle section (point 2), smartphones were placed there on the floor beneath the seat.

## 3. Results

### 3.1. Presentation of a Sample of Raw Results and Their Contents

The data obtained from the application were exported in CSV format. That data included timestamps of each measurement sample with an accuracy of up to one-thousandth of a second. For each measurement sample, acceleration values were recorded along the *x* (lateral), *y* (longitudinal), and *z* (vertical) axes. Additionally, the data included sound level, geographical coordinates, and vehicle speed. A sample of the exported data is presented in Table 1.

### 3.2. Discussion of the Data Processing Methodology and Extraction of Additional Parameters

**Figure 5:**
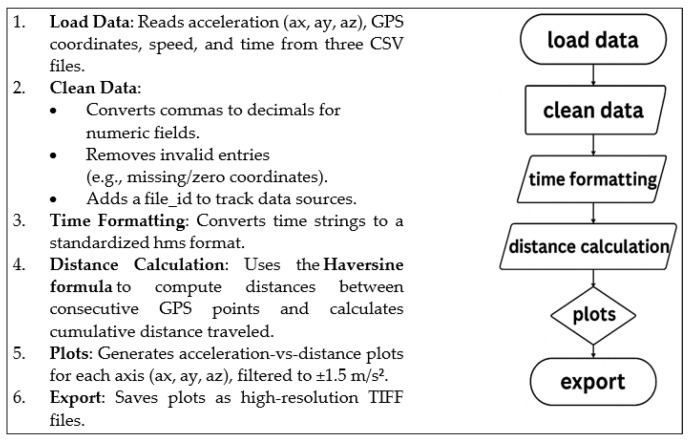
Algorithm for the analysis of raw measurement data.

The raw data were exported to text files (.txt). Data plots were generated using a script that processed GPS and accelerometric data from CSV files for the purpose of vehicle-motion analysis. The key steps included in Figure 5.

The Haversine formula mentioned above is one of two methods that can be used to calculate the distance between two points and, if the time is known, also to determine the speed over that distance. Both methods are described below:

1. Haversine method (considering Earth’s spherical shape):
  Uses the haversine_distance () function, which calculates the distance between two points on the surface of a sphere, accounting for the Earth’s curvature. This method provides greater accuracy over larger distances.
2. Simplified method (Cartesian approximation):
  Uses the simplified_distance () function, which approximates the Earth’s surface as a flat plane. This approach is computationally faster but less accurate, particularly over longer distances.

In both methods, the procedure for calculating distance follows the same general steps:

1. Iterate through consecutive pairs of GPS coordinates.
2. Calculate the distance between each pair of points.
3. Sum the distances to obtain the total (cumulative) distance of travel.

Once the distance has been calculated, speed can be determined using the following steps:

1. Calculate the time difference between consecutive GPS points.
2. Divide the distance by the time difference to obtain the speed.
3. Convert the speed to kilometers per hour (km/h).

### 3.3. Presentation of a Sample of Processed Data

**Figure 6:**
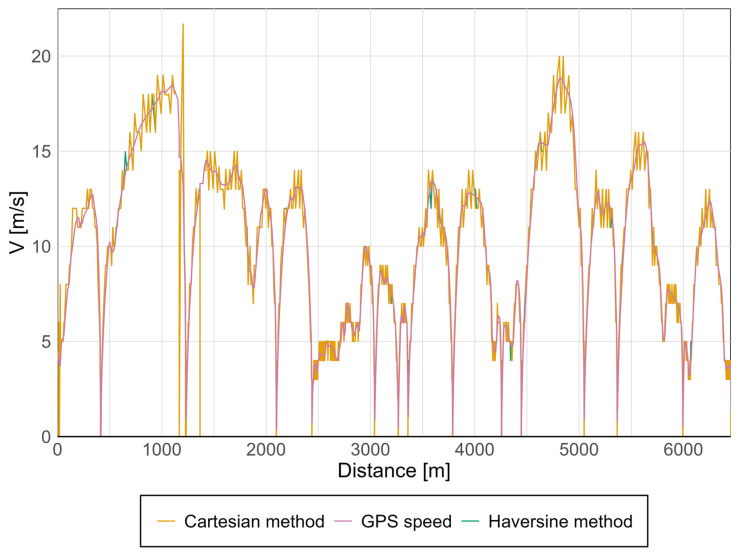
Speed graph presenting, in sequences, the results obtained from GPS measurements, followed by those calculated using the Cartesian and Haversine methods.

An example velocity graph calculated using both methods is presented in Figure 6. The most prominent feature is the velocity curve obtained with the Cartesian method, which is shown before the velocity curve calculated using the Haversine method [41].

As observed from the graph above, over a relatively short distance, approximately 7 km, there is no significant discrepancy between the two computational approaches. The curves virtually overlap across most of the route, leading to the conclusion that accounting for Earth’s curvature over such a short span has a negligible effect on the results. In essence, the Earth behaves as if it were flat over this distance.

It is noteworthy to compare the GPS-derived velocity to the calculated values. The GPS-measured velocity exhibits a relatively smooth profile with no abrupt fluctuations, whereas the computed velocity displays discontinuous jumps. This discrepancy may stem from the fact that the calculation methods yield average velocities over discrete segments—a limitation that becomes more pronounced as segment length increases, thereby introducing larger errors.

### 3.4. Presentation of Results in Graphs with Discussion and Comparison

**Figure 7:**
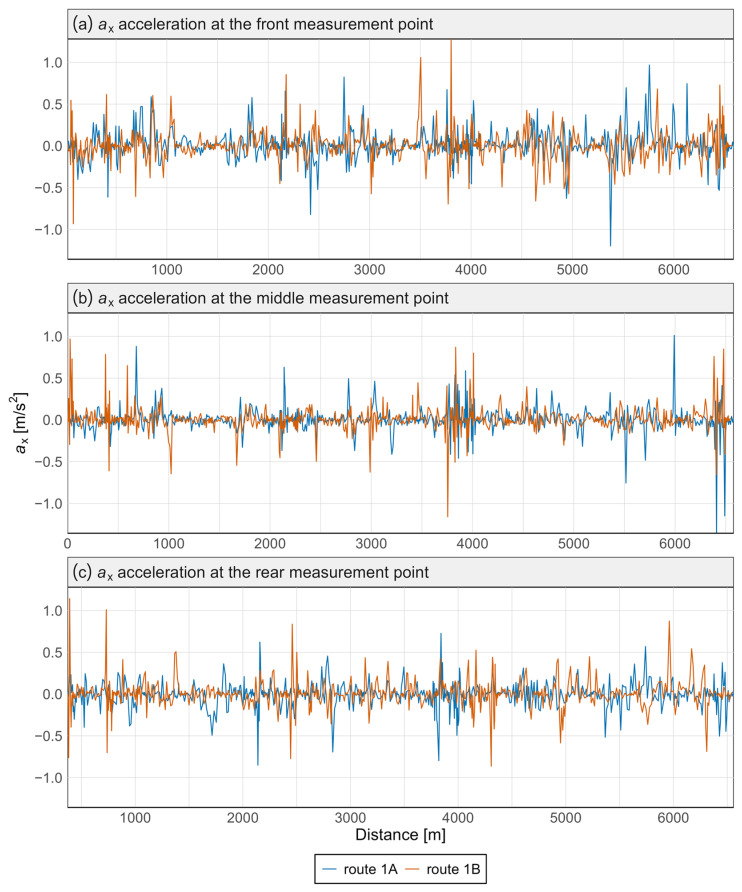
Acceleration graph along the *x*-axis in the Pesa 128NG tram at three measurement points.

**Figure 8:**
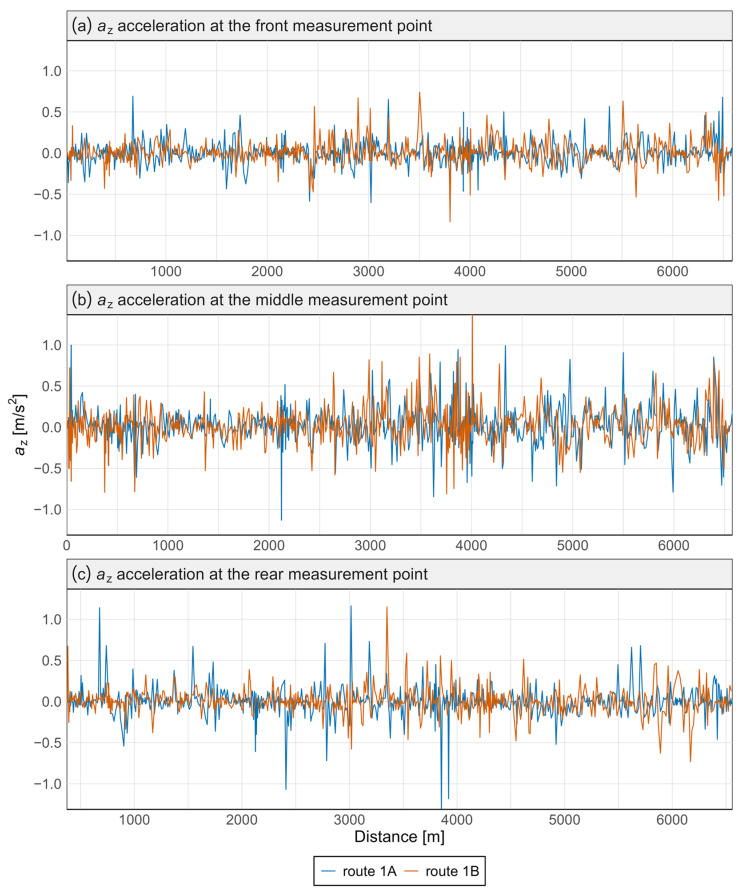
Acceleration graph along the *z*-axis in the Pesa 128NG tram at three measurement points.

Figure 7 and Figure 8 present the acceleration data along the *x* and *z* axes for the Pesa 128NG vehicle, measured at three locations: the front, center, and rear of the vehicle. Each measurement point is displayed on a separate graph. Distinct curves represent the route from Śródmieście SKM to Lawendowe Wzgórze (route 1A) and the return direction (route 1B).

Most observed values in Figure 7 and Figure 8 are close to zero, indicating standard operations without significant lateral or vertical interactions. The brief peaks may be associated with track irregularities in the vertical or lateral planes.

No significant qualitative differences were found between routes 1A and 1B—the two tracks almost completely overlap—indicating good reproducibility of driving conditions. This was confirmed by the Mann–Whitney and Kolmogorov–Smirnov tests, which revealed no statistically significant differences in acceleration distributions between the routes, either at specific points or along any axis (Table 2).

**Figure 9:**
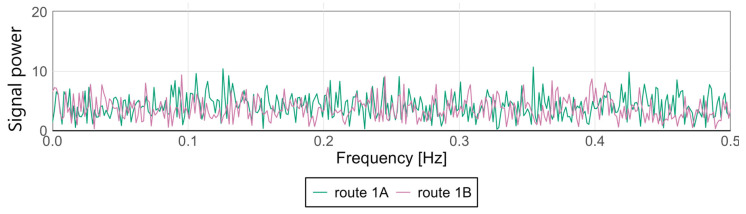
Spectral analysis of the *x*-axis at the middle point for the Pesa 128NG.

**Figure 10:**
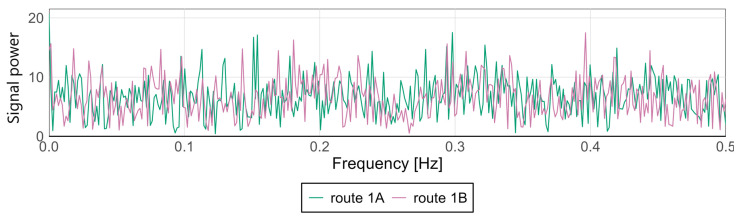
Spectral analysis of the *z*-axis at the middle point for the Pesa 128NG.

Variations in signal characteristics—particularly local extremes—may be more pronounced at the front or rear of the carriage, often as a result of train dynamics such as swaying or the “buoyancy” of the consist. This type of analysis enables rapid identification of nonlinearities and anomalies along a route section, allowing the localization of areas where track modernization or adjustments to traffic-management procedures are required. Figure 9 and Figure 10 present spectral analyses that further confirm these findings.

For the Pesa 128NG, most observed amplitude values at low frequencies remain minimal, indicating stable lateral and vertical acceleration levels during motion. Occasional peaks may correspond to recurring phenomena such as cyclic track irregularities, oscillatory motion of the traction unit, or structural resonances.

The spectra display highly similar patterns—both in distribution and amplitude—suggesting consistent conditions and technical uniformity between the two runs. The absence of dominant frequencies or significant deviations between routes indicates an even distribution of dynamic loads and effective infrastructure maintenance.

The data originate from a prepared measurement set, processed and cleansed of outliers, and synchronized over a common distance range. The implemented code ensures that the graph can be reproduced identically for subsequent measurement series or different routes. This visualization allows for a precise depiction of the dynamic behavior of the train set at various carriage points while maintaining the highest standards of data presentation.

**Figure 11:**
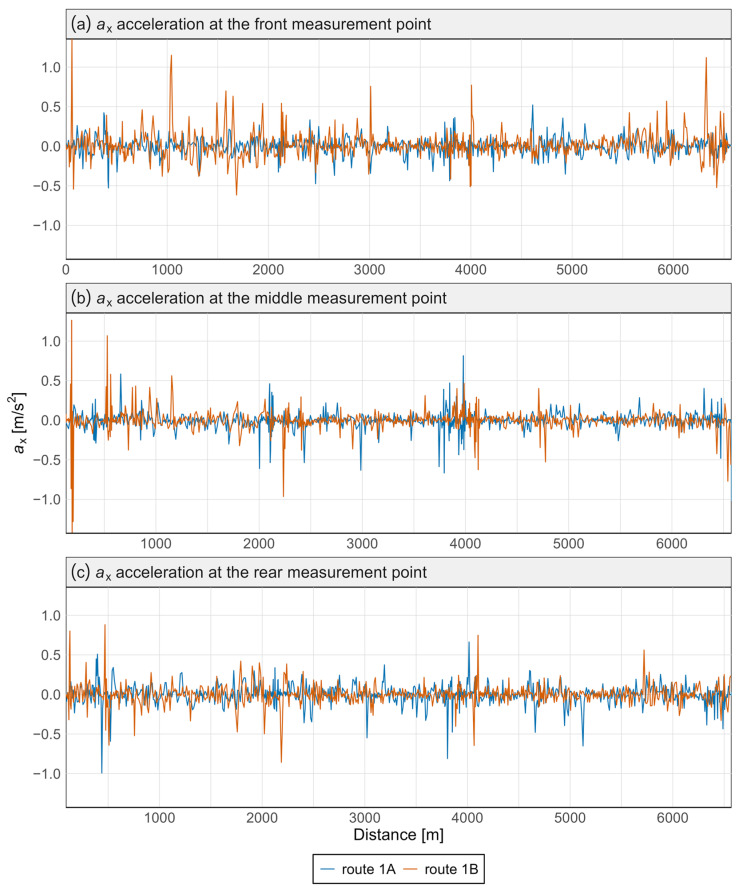
Acceleration graph along the *x*-axis in the N8C tram at three measurement points.

**Figure 12:**
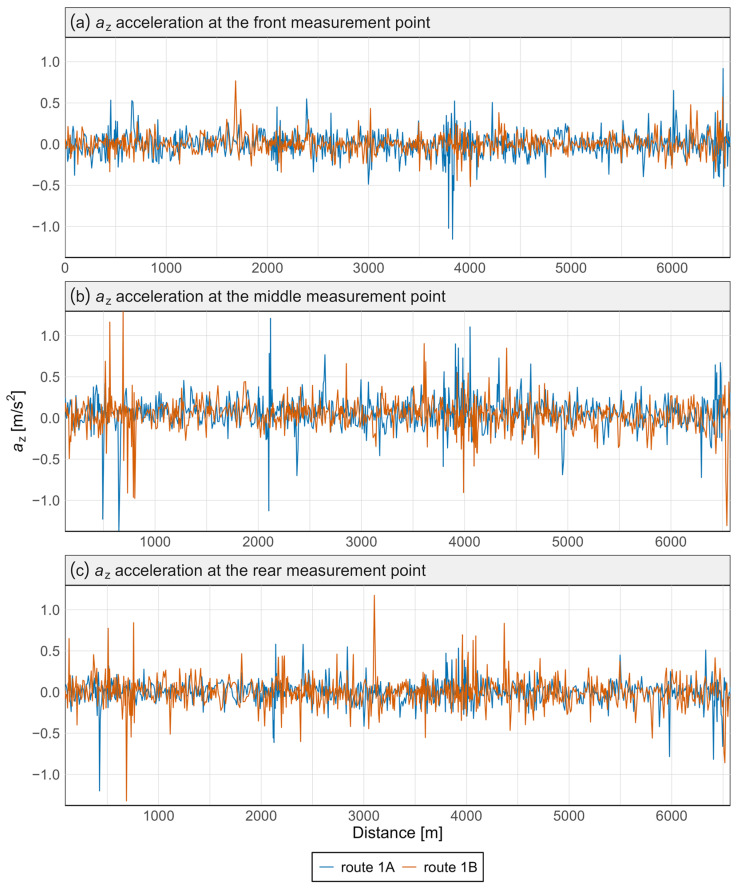
Acceleration graph along the *z*-axis in the N8C tram at three measurement points.

Acceleration graphs were also prepared for the N8C tram. These were developed in the same manner as for the 128NG tram, with each measurement point presented in a separate graph and both directions shown as separate curves. The results are presented in Figure 11 and Figure 12.

In the case of the N8C tram, the highest acceleration values along the *x*-axis (Figure 11) were also recorded at the front measurement point, confirming the possibility of the front wheelset climbing onto the rails on curves. Acceleration values in this axis are also elevated at the rear measurement point, with the lowest values occurring at the center of the vehicle.

In the *z*-axis (Figure 12), similar to the 128NG tram, the highest acceleration values for N8C occur in the central part of the vehicle, with lower values measured at the rear and then at the front. As with the Pesa vehicle, the *x*-axis data show comparable acceleration spikes at each measurement point, indicating consistent discomfort zones. In contrast, in the *z*-axis, such spikes are less evident, with only isolated peaks at various measurement locations.

**Figure 13:**
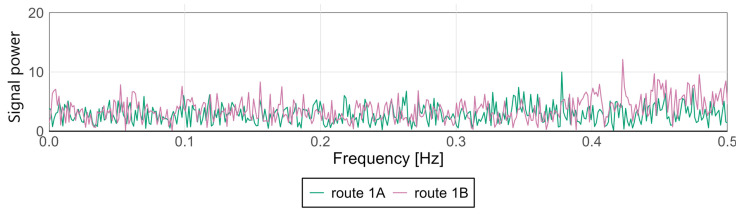
Spectral analysis of the *x*-axis at the middle point for the N8C.

**Figure 14:**
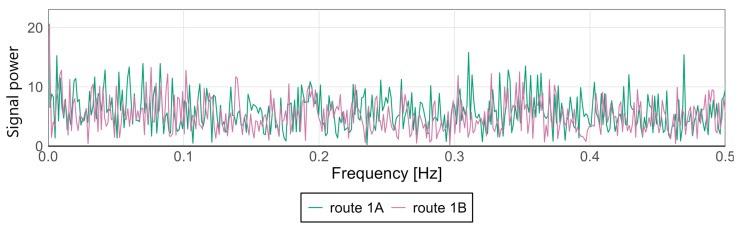
Spectral analysis of the *z*-axis at the middle point for the N8C.

Acceleration values in both directions of travel are generally similar, although isolated spikes appear only in one direction in some cases. For the Düwag N8C spectral analysis was also prepared. Results are shown in Figure 13 and Figure 14.

On the *x*-axis, both routes exhibit low, scattered amplitude values within the analyzed frequency range—no strong resonances are observed. Route 1B may be slightly more susceptible to small disturbances at higher frequencies compared to Route 1A, but the differences are not pronounced. The spectral analysis indicates that both routes are similar in terms of lateral and vertical accelerations within the analyzed frequency range. This is corroborated by the Mann–Whitney test (*p* = 0.538) and the Kolmogorov–Smirnov test (*p* = 0.847).

On the *z*-axis, both the Mann–Whitney U test (*p* = 0.003) and the Kolmogorov–Smirnov test (*p* = 0.025) indicate that, despite the apparent similarity of the curves, the distributions are statistically different. These results suggest that even if the average characteristics of the routes appear similar, statistical nuances point to differing vibration conditions—which may be significant for comfort assessment or component durability.

The plot does not reveal clear differences to the naked eye, but precise statistical analysis shows differences in the amplitude-spectrum distribution. The results justify using both visual inspection and statistical tests for evaluating vibrations and comfort, as the latter detect significant, non-random differences between routes. Statistical tests are more sensitive; they identify subtle, localized, or distributed differences in data distributions—such as the number and density of very small amplitudes or the presence of a few outliers—that are not visible visually. What may appear as noise to the human eye may constitute a significant population difference statistically.

This spectrum visualization reveals the general character of the signal and allows assessment of macroscopic differences (e.g., large peaks or shifts visible to the naked eye), while statistical tests capture small but repeatable distributional differences in large datasets via sensitive non-parametric methods.

**Figure 15:**
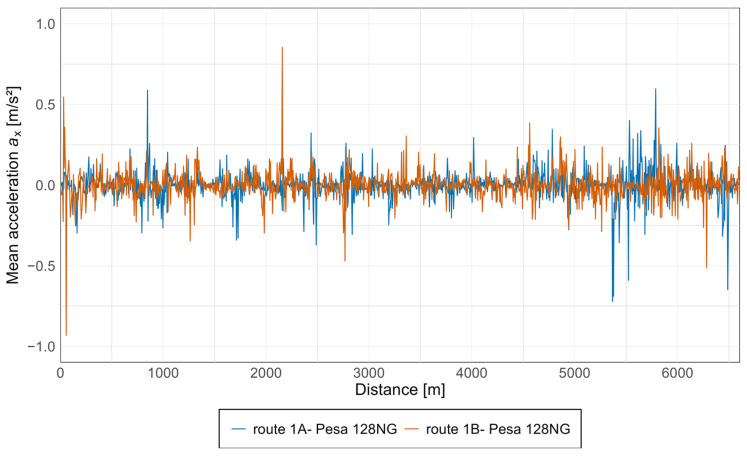
Average acceleration along the *x*-axis in the Pesa 128NG tram.

**Figure 16:**
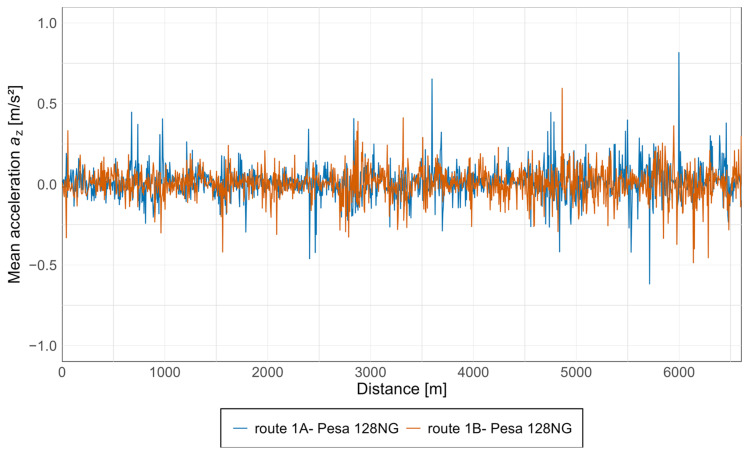
Average acceleration along the *z*-axis in the Pesa 128NG tram.

Subsequently, average acceleration values were calculated for all measurement points, enabling an assessment of the overall level of acceleration experienced by passengers in the vehicle. The results for the Pesa 128NG tram, in both the *x* and *z* axes, are presented in Figure 15 and Figure 16.

As can be observed from the above graphs, acceleration values in both directions are similar, with the highest values starting at approximately 5 km, particularly along route 1A, which corresponds to the oldest section of the track along Armii Krajowej avenue. Notably, based on the acceleration values along the *z*-axis, an increasing trend is evident during travel from the newest infrastructure toward the oldest, with isolated spikes occurring along each section.

**Figure 17:**
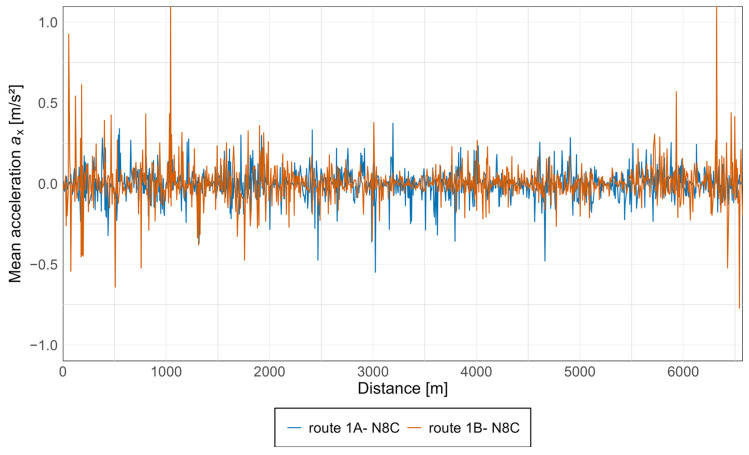
Average acceleration along the *x*-axis in the N8C tram.

**Figure 18:**
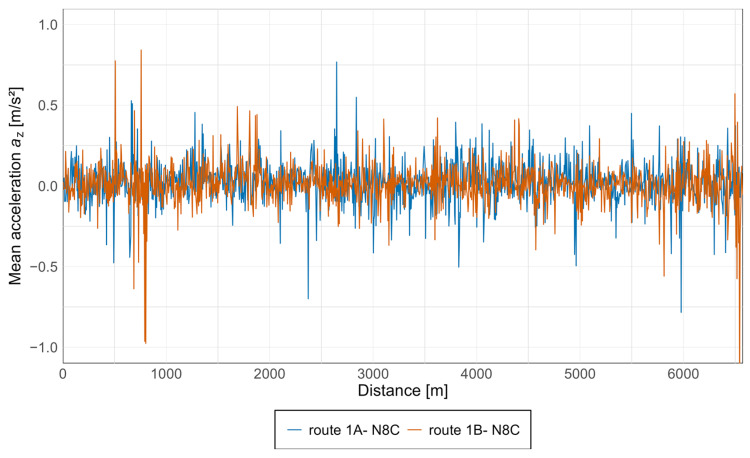
Average acceleration along the *z*-axis in the N8C tram.

Equivalent graphs were prepared for the N8C tram, as shown in Figure 17 and Figure 18 below.

Along the *x*-axis in the N8C vehicle, an opposite trend to that observed in the Pesa 128NG can be noted. The highest acceleration values occur on the newest infrastructure, with the lowest values recorded on the intermediate infrastructure. Notably, acceleration peaks appear along route 1B at the beginning and end of the track section, which may indicate poorer track conditions in the direction of Lawendowe Wzgórze.

In the *z*-axis, acceleration values remain high throughout the entire length of the route, with extreme values similar to those seen in the *x*-axis accelerations at the start and end of the segment.

**Figure 19:**
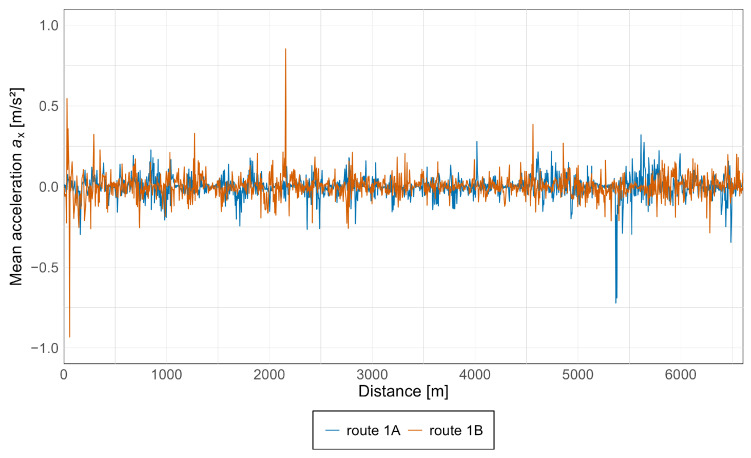
Average acceleration along the *x*-axis for both vehicles.

**Figure 20:**
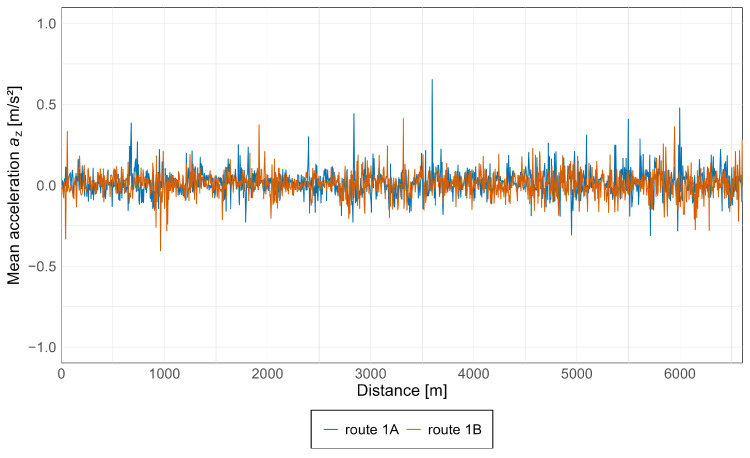
Average acceleration along the *z*-axis for both vehicles.

Additionally, graphs showing average acceleration values along the *x* and *z* axes for both vehicles were prepared, as presented in Figure 19 and Figure 20.

As can be observed above, the largest fluctuations in unbalanced acceleration values along the *x*-axis occur near the Lawendowe Wzgórze stop, while along the *z*-axis they appear near Śródmieście SKM stop. This indicates greater acceleration sensations in the *x*-axis on newer infrastructure, and in the *z*-axis on older infrastructure.

**Figure 21:**
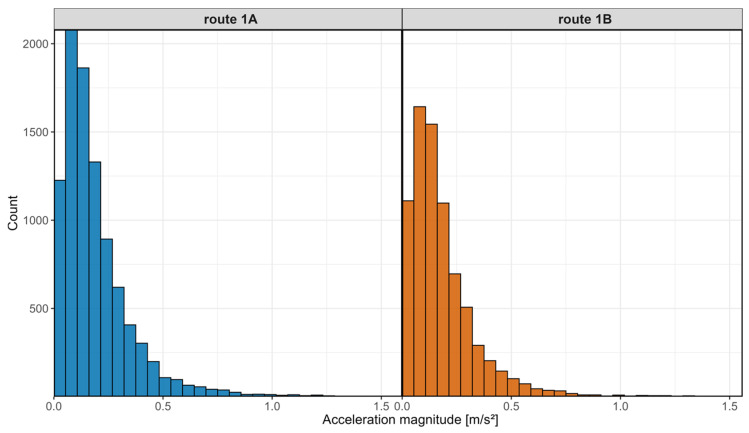
Acceleration histogram.

To illustrate the distribution of accelerations along the route in both directions, histograms were prepared and are presented in Figure 21.

The above histograms display the number of acceleration samples recorded during the measurements on the vertical axis, and the acceleration values on the horizontal axis. Based on these histograms, it can be observed that the acceleration values most frequently fall within the range of approximately 0.05 m/s^2^ to 0.15 m/s^2^. Extreme values reaching up to around 1 m/s^2^ occurred only in exceptional cases. The histograms exhibit a Gaussian curve shape with a median value close to zero.

**Figure 22:**
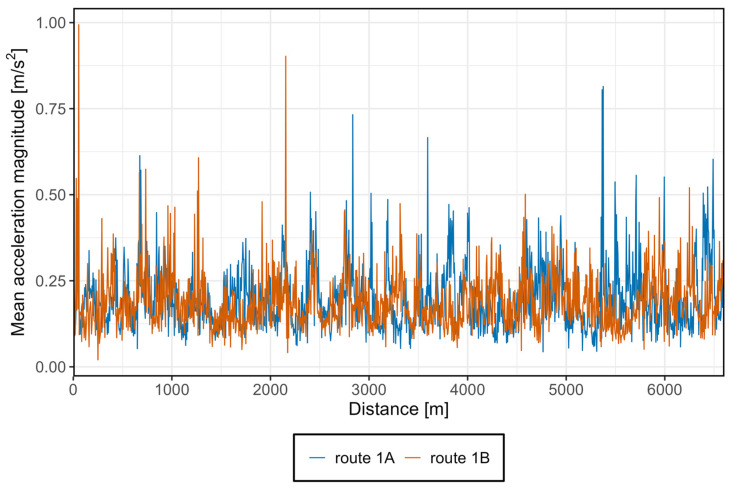
Average acceleration without distinguishing axis.

To represent the total acceleration experienced by passengers, a graph was prepared showing the average acceleration regardless of axis or vehicle type but distinguishing the direction of travel. The results are presented in Figure 22.

Based on the graph, it can be observed that passengers are subjected to a continuous acceleration of at least 0.1 m/s^2^. During the journey, significant acceleration spikes occur in both directions. For direction A, this happens near Śródmieście SKM section, while for direction B, it occurs near the Lawendowe Wzgórze section. For most of the route, accelerations fluctuate between 0.1 m/s^2^ and 0.3 m/s^2^, which confirms the observations from the above histogram.

The graph also shows that acceleration values in direction A do not fully coincide with those in direction B. This discrepancy may be due to differing infrastructure conditions, smaller curve radii on the inner track compared to the outer track-which is more noticeable on tram infrastructure than on railways due to curves-or differences in braking and acceleration points near stops, causing shifts relative to the opposite track.

**Figure 23:**
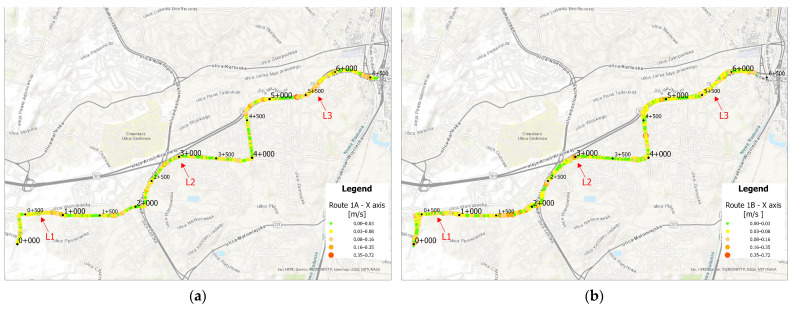
(**a**) Average acceleration values along the *x*-axis on route 1A, (**b**) Average acceleration values along the *x*-axis on route 1B.

**Figure 24:**
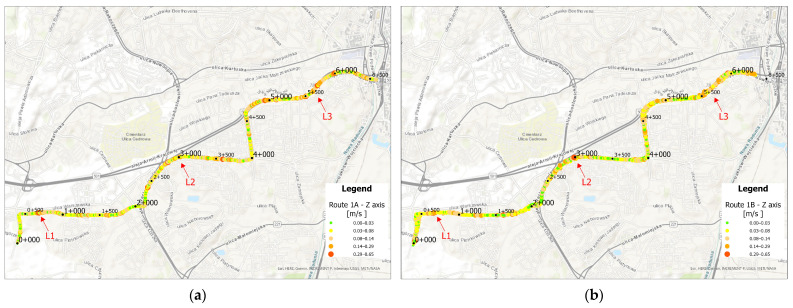
(**a**) Average acceleration values along the *z*-axis on route 1A, (**b**) Average acceleration values along the *z*-axis on route 1B.

Using the previously presented data, average acceleration values along the *x* and *z* axes for an averaged vehicle on route 1 were visualized in ArcGIS. The results are shown in Figure 23 and Figure 24. Based on the visualizations below, it can be observed that a GPS measurement error occurred along route 1B, causing a slight misalignment of points within a single-track path. However, this does not significantly affect data interpretation.

Along the *x*-axis, higher acceleration values can be observed along Armi Krajowej avenue, where the trams are able to accelerate to the maximum permitted speed due to long distances between stops. Additionally, traveling through curves on older infrastructure may result in increased acceleration readings. Elevated values are also noticeable on the newest section of the route in curved areas where the speed limit is 70 km/h. Furthermore, acceleration peaks appear at various curved segments along the route in both directions.

Along the *z*-axis, higher acceleration values are particularly visible in curved sections, occurring consistently throughout the entire route. In direction A, curved segments on the newly constructed track are especially noticeable, where high permitted speeds are maintained and acceleration is recorded despite the relatively low curvature compared to other sections.

**Figure 25:**
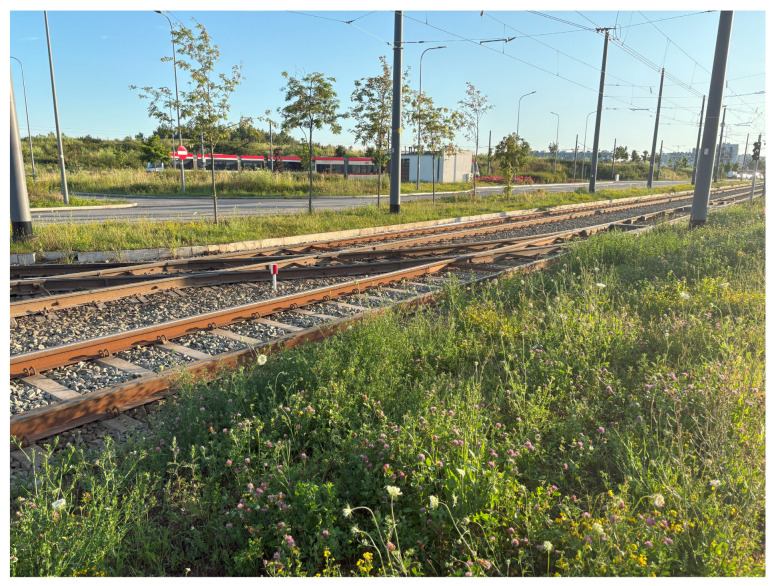
Location 1—Ujeścisko node with acceleration peak along the *z*-axis.

Based on the maps above, locations with acceleration peaks were identified and photographed. The first peak, shown in Figure 25, occurred at the crossing panel in the Ujeścisko junction. The layout of this junction places the turnout approximately 200 m from the tram stop, preventing trams from reaching full operating speed—and tram drivers often do not adhere to the 15 km/h restriction required when using shallow flanges. Consequently, overspeeding leads to higher vertical (*z*-axis) accelerations, reflecting the tram bumping on the crossing panel.

**Figure 26:**
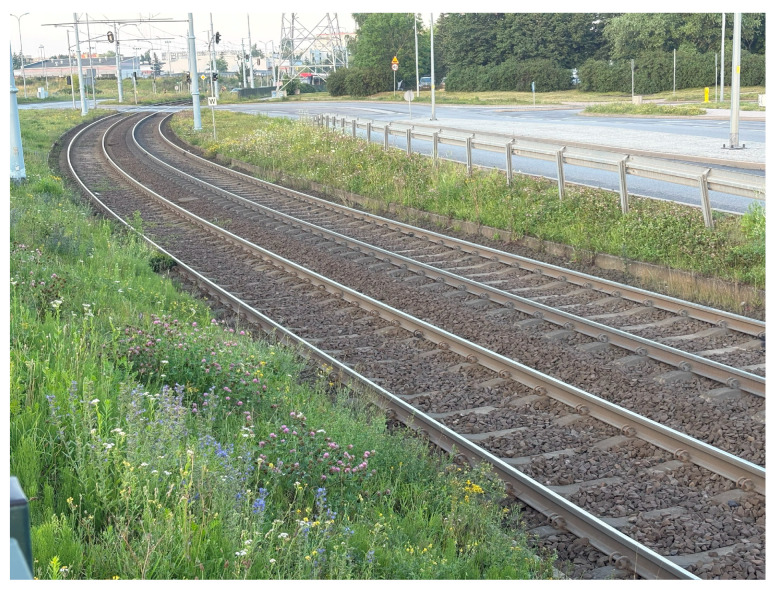
Location 2—Curve between Cebertowicza and Wilanowska tram stops with acceleration peaks.

The second acceleration peak occurred on the curve between the Cebertowicza and Wilanowska tram stops (Figure 26). An insufficient layer of track ballast was observed, which may have compromised the curve’s design geometry. This issue could be exacerbated by the transition from 49R1 rails on the curve to 60R2 rails at the crossing, as the two rail profiles have different stiffness. The resulting variable radius may generate acceleration peaks throughout the curve.

**Figure 27:**
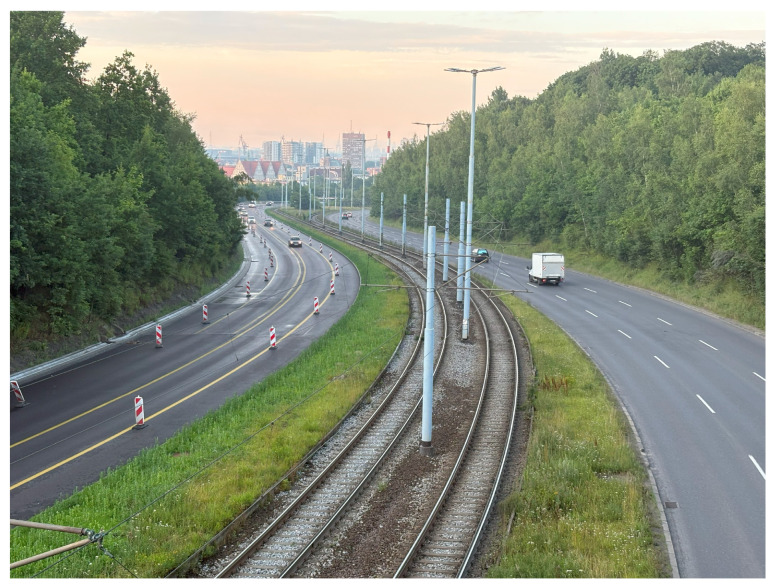
Location 3—Curves between Odrzańska and Pohulanka tram stops with acceleration peaks.

The third acceleration peak was observed along the track on Aleja Armii Krajowej (Figure 27). Between the Odrzańska and Pohulanka stops, the alignment runs for approximately 800 m adjacent to a high-speed roadway, allowing tram operators to build up speed through curves designed to match road geometry. These curves may have been engineered for lower speeds, resulting in acceleration peaks when traversed at higher velocities.

## 4. Conclusions and Discussion

The conducted research confirmed that mobile technologies based on smartphones equipped with MEMS sensors and GPS modules can serve as a reliable and cost-effective alternative to traditional tram dynamics monitoring systems. Conventional solutions, which rely on expensive, high-precision accelerometers and dedicated software, are often inaccessible to many tram network operators. Measurement of lateral (*x*) and vertical (*z*) accelerations, carried out using the Physics Toolbox Sensor Suite Pro application at three locations within Pesa 128NG and Düwag N8C trams, demonstrated sufficient accuracy and repeatability to support the preliminary identification of track segments requiring increased attention and detailed investigation, including on-site inspection, regarding the condition of the surface structure and track geometry. Non-parametric tests (Mann–Whitney U and Kolmogorov–Smirnov) showed no significant differences between repeated runs on most axes, confirming the reproducibility of the measurement conditions.

Our analysis confirmed that omitting longitudinal accelerations—which are strongly influenced by the vehicle’s operational style—does not diminish the diagnostic value of lateral and vertical measurements. This enables a simplification of the measurement procedure without compromising data quality. The observed acceleration distribution resembled a Gaussian profile, with the most frequently occurring values in the range of 0.05–0.15 m/s^2^ and extreme readings reaching approximately 1 m/s^2^. These results are consistent with classical studies on rail vehicle vibrations, including those conducted by others. Spectral analysis did not reveal dominant resonant peaks, supporting the stability of both vehicle types across the measured frequency bands.

The reliability of velocity calculations was confirmed by comparing results obtained using the haversine formula and a Cartesian approximation. Over a distance of approximately 7 km, both methods produced nearly identical results. Data were visualized as georeferenced acceleration maps in ArcGIS, clearly highlighting “hot-spot” segments along two routes.

Combining triaxial accelerometric data with synchronized GPS positioning enabled the development of georeferenced acceleration maps. These spatial distribution maps clearly highlighted route sections with degraded track quality—particularly, the segment along Al. Armii Krajowej and the areas near the Śródmieście SKM and Lawendowe Wzgórze stops. Such maps facilitate the prioritization of maintenance tasks and enhance infrastructure management.

The main limitation of the proposed approach remains the necessity to calibrate smartphone measurements against data from certified instruments—such as piezoelectric accelerometers used in accredited test benches—and to establish clear guidelines for sensor installation within the vehicle. Inter-device variability, caused by different smartphone models (e.g., Galaxy S10e, iPhone 14 Plus, Xiaomi Mi 10T Lite 5G) and mounting locations (bogie-area shelf vs. sandbox beneath seats), and non-uniform sampling intervals (0–20 ms) must be addressed through procedural standardization and potential software or firmware harmonization. Inter-device variability and mounting configurations can affect the accuracy of recorded values, making procedural standardization essential. Additionally, the adopted method of averaging values can be used to identify track segments requiring greater attention, but it is not suitable for analyzing individual rolling stock. In such cases, analysis should be performed on a filtered group of runs for a single tram model.

In future work, we will focus on three key areas:

- validating smartphone-derived data against certified sensors to quantify errors and refine data analysis algorithms,
- incorporating longitudinal acceleration into the evaluation to assess passenger stability during acceleration and braking,
- addressing the challenge of non-uniform MEMS and GPS sampling frequencies through potential firmware or app-level enhancements.

Furthermore, tests on trams operating on different track gauges (1000 mm and 1435 mm) are planned to examine how infrastructure geometry influences acceleration patterns. The ultimate goal is to develop a multi-criteria comfort index and propose a real-time monitoring system deployable under operational conditions.

In conclusion, the pilot study demonstrated that a low-cost MEMS and GPS monitoring system can become an integral component of tramway infrastructure maintenance. With proper calibration and standardized procedures, this solution has the potential to support effective network management and improve passenger ride comfort. In the future, the low-cost smartphone-based system will not only enable the assessment of ride comfort in accordance with the methodology outlined in PN-EN 12299:2009 but also provide real-time alerts accessible to tram operators via onboard mobile devices. This can assist in adjusting driving techniques, enhancing ride smoothness and safety. Simultaneously, the methodological simplicity of the mobile solution facilitates its widespread deployment, especially in networks with limited budgets.

## Figures and Tables

**Table 1:** Raw accelerometric data collected from onboard vehicles.

Time [hh:mm:ss]	Acceleration *ax* [m/s^2^]	Acceleration *ay* [m/s^2^]	Acceleration *az* [m/s^2^]	Gain [dB]	Latitude [°]	Longitude [°]	Speed [m/s]
…	…	…	…	…	…	…	…
09:13:11:005	−0.0392	0.0905	0.0089	97.8385	54.35	18.643	4.46
09:13:12:006	−0.0343	−0.2142	0.1676	99.1421	54.348	18.643	4.28
09:13:13:007	−0.7291	−0.1872	−1.2267	99.7207	54.348	18.642	3.91
09:13:14:007	−0.3037	−0.1197	−0.1439	97.9997	54.348	18.642	4.07
09:13:15:007	−0.295	−0.0176	0.0253	99.7355	54.348	18.642	3.93
09:13:16:009	0.5498	−0.0033	−0.9949	99.9674	54.348	18.642	3.8
09:13:17:009	−0.2679	0.1003	0.5391	99.6387	54.348	18.642	3.72
…	…	…	…	…	…	…	…

**Table 2:** Mann–Whitney test and Kolmogorov–Smirnov test results for Pesa 128NG.

Axis	Point	*p*-Value Mann–Whitney Test	*p*-Value Kolmogorov–Smirnov Test	Significant Difference
*a_x_*	Front	0.774	0.387	none
*a_x_*	Middle	0.721	0.875	none
*a_x_*	Rear	0.137	0.232	none
*a_z_*	Front	0.871	0.891	none
*a_z_*	Middle	0.249	0.232	none
*a_z_*	Rear	0.300	0.390	none

## Data Availability

The data set used in the research can be found by the DOI https://doi.org/10.34808/97x4-df39.

## Abbreviations

The following abbreviations are used in this manuscript:

MEMS	Microelectromechanical Systems
GPS	Global Positioning System
IMU	Interrail Measurement Unit
SKM	Fast Urban Railway
DC	Direct Current
EMU	Electric Multiple Units
DMU	Diesel Multiple Units
